# Effects of Residential Instability of Renters on Their Perceived Health Status: Findings from the Korean Welfare Panel Study

**DOI:** 10.3390/ijerph17197125

**Published:** 2020-09-29

**Authors:** Kiduk Park, Wonseok Seo

**Affiliations:** 1 School of Public Service, Chung-Ang University, Seoul 06974, Korea; kpark0201@gmail.com; 2 Department of Urban Planning and Real Estate, Chung-Ang University, Seoul 06974, Korea

**Keywords:** perceived health, mental health, physical health, residential instability, renters

## Abstract

Identifying the impact of housing instability on the health status of renters with relatively high economic difficulties is important for the improvement of renters’ quality of life and their social security. Accordingly, this study adopted a panel data regression approach to examine the associations between residential instability and perceived health status—including physical and mental health—using 14 waves (2006–2019) of longitudinal data collected by the Korean Welfare Panel Study. The results showed that residential instability significantly affected perceived health status, and renters who experienced residential instability perceived worse health status and had more severe depression than those who did not experience residential instability. Moreover, failure to meet the minimum housing standard worsened depression in renters. Despite assistance benefits from the government, permanent rental housing and the national basic living security were also factors that worsened depression. Dissatisfaction with one’s residential environment and social relationships were also associated with increased depression. We recommend that the overall quality of housing welfare services, including a focus on the mental health of low-income renters, be improved by expanding the range of services, increasing the number of professional housing welfare workers, and supplying community facilities for increasing residential and social relationship satisfactions.

## 1. Introduction

One goal of housing policy is to ensure residential (housing) stability for socially vulnerable groups who do not reside in a residential environment that is above a certain quality level. In general, the lower the income, the poorer the residential environment and the higher the residential instability. While most homeless people experience a period of residential instability before becoming homeless [1], the concept of residential instability is quite different from homelessness. People who experience residential instability may have a place to reside but experience various ongoing challenges related to keeping the residence. Residential instability is frequently used as an umbrella term encompassing a range of housing-related issues, and researchers have interpreted and measured residential instability using different approaches [2]. These include difficulty paying rent, frequent residential moves, eviction threats or notices, or “doubling up” with friends or family [3,4,5]. In this study, we define the concept as an experience shaped by two negative forms of housing-related problems due to financial hardships: (1) overdue rent and (2) eviction. These refer to situations wherein the renter is unable to pay their rent for a certain period or involuntarily moves out due to their economic situation.

While the potential negative effects of residential instability are abundant, one critical consideration is its relation to health. People experiencing residential instability are more likely to have lower access to healthcare facilities and lower rates of proper hospital utilization than those with stable housing [6]. Studies have reported poor health status among populations experiencing various types of residential instability, from homelessness [7] to foreclosure [8]. After the Korean War, Korea implemented growth-oriented policies for rapid national reconstruction, thereby achieving high economic growth. However, this process resulted in social side effects such as a high economic gap and an increase in the number of underprivileged people. Today, pressing social problems, such as unsolicited crimes due to economic alienation and lonely death due to social isolation, are prevalent, and residential instability among the underprivileged is regarded as an important cause [9]. Residential instability among the underprivileged also has a negative impact on their physical and mental health, causing their quality of life to deteriorate. In particular, renters, who are more economically alienated than owner-occupied residents, are the core population of those affected by residential instability, and the perceived health status of renters has a significant impact on society. In this regard, the impact of residential instability on the health of the Korean underprivileged—primarily low-income renters—is an important and urgent research topic to enhance social stability as well as to improve quality of life for this population.

Studies on residential environment and health-associated variables in Korea to date are growing in number; however, their range remains limited overall. Research in Korea to date has primarily focused on the relationship between the physical condition of the residence (minimum housing standards) or the allocation of family income to housing cost (housing affordability) and residents’ health outcomes, rather than identifying the overall relationship between residential instability and the health status of renters [10,11]. In addition, most studies on residential instability have been particularly restricted in generalizability, since they have primarily focused on analyzing merely a small fraction of low-income households. Thus, there is a need to investigate the association of residential instability with poor health and social outcomes in Korea with representative panel data. To address this gap, the present study adopted the panel data regression approach to investigate the associations between residential instability and perceived health status—including physical and mental health—using data from the Korean Welfare Panel Study (KoWePS). In addition, this study included residential security, welfare programs, residential environment, and individual characteristics as independent variables, considering that residential instability may mediate the relationship between these factors and health outcomes. Furthermore, a comparison between the determinants of perceived physical and mental health status identified in this study would provide guidelines to effectively improve low-income households’, specifically renters’, quality of life.

## 2. Literature Review 

Residential instability might be a potential indicator of homelessness. It includes a broad spectrum of housing-related problems, such as being severely cost-burdened, living in overcrowded or doubled-up residences (i.e., living with friends or family to decrease costs), and frequent residential moves, all of which low-income renter households may experience before becoming homeless [3,12,13]. The concept of residential instability is commonly used to indicate housing situations among those struggling just before homelessness. Thus, households that experience residential instability experience a spectrum of housing-related issues that are more severe than those with housing affordability problems (usually allocating 30% of household income on housing cost). They tend to be more economically disadvantaged than the overall poverty population and are more likely to experience homelessness at some point [14].

Research suggests that the association between housing instability and health status or health behavior is closely related to loss of housing, overcrowding experienced by those who have to live in shared housing to save money, and residential mobility. Some studies, for instance, suggest that housing loss causes mental illness because it decreases one’s feeling of personal control and induces stress [15,16,17]. Negative health behaviors likely associated with stress from housing instability include drug abuse and unhealthy eating habits, which can lead to chronic diseases [18]. In addition, frequent residential moves may disrupt important informal social networks with neighbors, kin, and friends, which makes it difficult to form social ties with neighbors and, in turn, diminishes social support. These also include compromising on meaningful resources, such as healthy eating and shopping choices, safe spaces to exercise, and trusted healthcare providers [3]. Additionally, moving to low-cost housing may come with more hazardous or unsafe living conditions.

However, while severe housing cost burden may increase stress, it does not usually include some of the negative effects that entail residential moves. The severe stress regarding housing payments and threat of impending eviction may be detrimental even to those who are not forced to move yet. Those with overdue housing costs may even compromise on healthcare, including time and cost for accessing healthcare services and following a healthy diet, to save extra money [19]. In addition, households with children are more likely to hesitate to live in homeless shelters or places not meant for sleeping (e.g., streets) due to safety concerns for their children. Thus, households with children tend to double up or live in extremely unstable residential situations. For these reasons, it is important to consider the housing situations of renters who are at imminent risk of eviction or have had recent moves for a better understanding of residential instability.

Several studies have investigated the relationship between residential instability and health and have revealed a close relationship between a resident’s economic status—indicated by factors such as income level—and their health status [3,20,21,22,23]. Studies have also shown that an economically disadvantaged status could cause depressive symptoms and lead to suicide and other psychiatric disorders [17,24,25,26]. Davey-Rothwell et al. [13] examined the minutely classified associations between residential mobility, economic status, and depressive symptoms; they found that people experiencing residential instability due to their economically disadvantaged status tend to move frequently and exhibit high levels of depressive symptoms regardless of gender. However, the level of depressive symptoms has been shown to be higher in women than in men, and these results are in keeping with previous studies by Nolen-Hoeksema et al. [27] and Weissman and Klerman [28]. Residential instability is also known to be an important factor causing the deterioration of the health status of children and adolescents, more so than that of other age groups [29,30,31]. However, health outcomes can be improved if living conditions for children and adolescents improve, such as living in public housing [32]. In accordance with this view, Desmond et al. [33] considered residential instability to be a significant factor for health deterioration. 

Howden-Chapman et al. [34] and Hiscock et al. [35] investigated the health status of people by examining the association between participants’ economic status and their housing situation. Specifically, these studies compared owner-occupiers with renters and found that being an owner-occupier is more advantageous to one’s mental health than being a renter. In the case of renters, they are unable to reside in owner-occupied housing with high residential stability due to insufficient funds. As a result, renters experience severe residential instability, which has a negative impact on their health; therefore, it could be suggested that the mental health of owner-occupiers is better than that of renters [33]. Subramanian et al. [36] analyzed residential stability and minutely classified income levels to examine their relationship with self-rated health. The results showed that people with higher levels of residential stability rated their health better, and those in the highest income bracket believed themselves to be more than twice as healthy as those in the lowest income bracket. As such, it could be understood that housing situations and financial stability play an important role in people’s health. 

Previous studies have shown that public rental housing could improve not only the residential stability of low-income renters but also their health [23,37,38]. This indicates that housing assistance could improve the quality of life for low-income renters. 

In Korea, studies have examined the relationship between people’s housing problems and their health status from multiple perspectives. These studies have confirmed that the level of depressive symptoms is generally higher among those who are impoverished, senior citizens, or renters, as the probability of these groups experiencing residential instability is higher due to their lower income status [9,39,40,41,42]. Further, another related study [11] showed that disadvantaged groups living in houses that are below minimum housing standards perceive their health to be poor as a result of their inadequate housing conditions and confirmed the negative impact of poor housing conditions on their life satisfaction.

Through an empirical analysis, Kang and Seo [9] examined the effect of housing situations on residents’ levels of depressive symptoms and concluded that the levels decrease as satisfaction with economic status increases and that the level of depressive symptoms is higher among renters than among owner-occupiers. Han and Jun [43] divided residents into two groups by housing situation—public rental housing and regular housing—and compared the mental health of their inhabitants. Their results were similar to that of Kang and Seo [9]; they found that the quality of mental health was lower in residents of public rental housing. Lim and Jang [44] categorized residents into three groups—owner-occupiers, long-term rent with lump-sum deposit (jeonse in Korean) renters, and monthly renters—and compared the mental health of each group. Their results showed higher levels of depressive symptoms and lower life satisfaction in the monthly renters group compared to the other groups. These findings suggest that housing assistance is necessary for monthly renters, as they reside in homes with poor housing conditions due to their limited ability to afford housing expenses. Park [45] also categorized residents into two groups—private renters and public housing residents—and examined their subjective health status through comparative analysis. Their results showed that permanent public housing residents with the lowest average income level had a comparatively poor health status. 

Based on the aforementioned studies, it is possible to suggest that residential instability negatively affects low-income groups’ health status from multiple perspectives. While some studies have indirectly examined the relationship between health status and residential mobility in economically disadvantaged renters, few studies have directly examined the relationship between renters’ residential instability and health status. As confirmed in previous studies, economically disadvantaged people from low-income groups exhibit high levels of depressive symptoms that could increase the risk of suicide. Accordingly, it could be argued that understanding the effect of financial difficulties on renters—who are at a greater risk of experiencing such difficulties—is important to improve not only renters’ quality of life but also their social security. Therefore, to address the gaps identified in the previous literature, as well as to consider policy changes for the improvement of renters’ quality of life and social security, the present study aimed to identify the effect of residential instability on the subjective physical health status and mental health of renters.

## 3. Materials and Methods

### 3.1. Data Collection

This study used 14 waves (1–14) of longitudinal data collected by the KoWePS between 2006 and 2019 to investigate the effects of residential instability on perceived health status, including the subjective physical and mental health (depression) of renters. An ongoing longitudinal study by the Korea Institute for Health and Social Affairs has been conducted on a nationally representative sample of Korean citizens over the age of 15 years since 2006. The survey sample represents 90% of the census conducted in the first survey, which may reflect the overall health status of the population in Korea. In addition, since about 50% of the total sample is selected from the low-income group (less than 60% of the national median income at the time of sampling), the data were especially appropriate for low-income targeted policies and poverty research.

**Figure 1:**
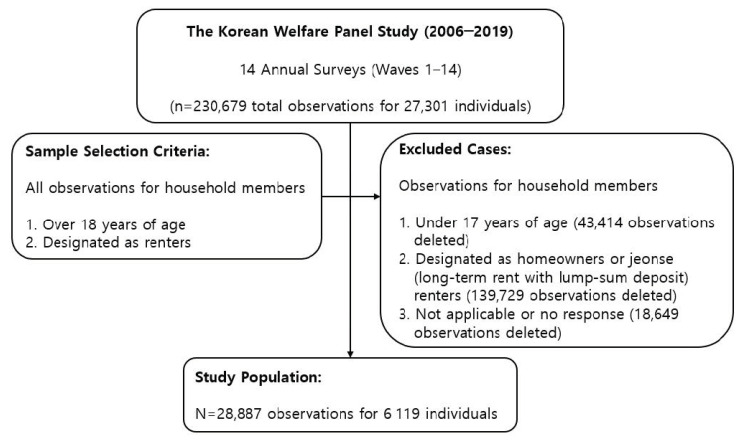
Description of the analytic sample selection.

The analytic sample selection is described in Figure 1. All KoWePS survey respondents living in rental housing and over the age of 18 years were selected, which yielded a sample of 28,887 from a total of 230,679 observations to be utilized for the empirical analysis. If the above conditions were met, we included all the observations of the panel data, even if some of those individuals dropped out in the middle of the waves. Therefore, panel analysis was performed using unbalanced panel data.

To consider respondents’ physical, mental, and social health, as defined by the World Health Organization, the analysis was conducted to explain the subjective health status and depression index of the participants. Studies have identified an association between subjective health status and mortality, chronic illness, hospital visits, and hospitalization [46,47,48]. Overall, self-rated health has been used a dependable tool to measure social inequalities in regard to health. In this study, perceived health status including subjective physical health and mental health can be used as a representative index to assess respondents’ overall health level. Specifically, subjective physical health was measured using a 5-point Likert scale, with respondents rating items about their physical health from 1 (very poor health) to 5 (excellent). In the assessment of mental health, the overall depression index was calculated by summing the scores on all items relating to depressive symptoms experienced by household members over the past week (see Table 1). Two positively worded items were reverse-coded: (b) “It was relatively good” and (g) “I was without major dissatisfaction”. Higher scores represented a higher level of depression.

Residential instability, the core independent variable of this study, was measured based on whether respondents had experienced “overdue rent for two months or more or relocation because they could not pay their rent”. If the house was owner-occupied or did not involve rent payment for the entire year, they were not considered as being residentially unstable and were excluded.

The minimum housing standards in Korea comprise three categories, which are defined by the Housing Basic Act: (1) size, (2) essential facilities, and (3) “structural”, “performance”, and “environmental” standards. Specifically, according to Article 2 of the Minimum Housing Standard (2011), the area of the house should range from more than 14 m^2^ (for one person) to 55 m^2^ (for six persons) in terms of household composition and the number of rooms. Essential housing facilities include flush toilets, bathroom, kitchen, and sewer facilities (Article 3). The minimum housing standards also include structural, performance, and environmental criteria for safety and comfort (Article 4) [49]. In this study, we included a binary variable that measured whether a household member (individual) lived in a house that did not meet the minimum standards based on any of the three criteria (*minimum housing standard*). In the empirical analysis, we also included three dummy variables that measured whether a household member participated in any government welfare programs: (a) whether a household lives in *permanent rental housing* and whether a household receives (b) *national health insurance* and (c) *national basic living security*.

To investigate possible causal associations between independent variables and health-related variables, all independent variables based on theoretical reasoning and previous studies were included (see Table 2). We included a dummy variable that measured whether a household lives in poor or *non-residential housing* (basement, semi-basement, rooftop housing, green house, shed, or shanty house) environments to examine the possible positive effect of improving access to better housing locations and conditions. To estimate whether the health-related variables are mediated by regional classification, a variable *metropolitan area* (whether living in a metropolitan area) was also included. Furthermore, to control for respondents’ perception of their residential environment, the *resident satisfaction* and *social relationship satisfaction* of household members were rated (1 = very dissatisfied to 5 = very satisfied) to assess the overall quality of the housing and neighborhood. To obtain consistent and unbiased estimates of the variables, time-varying factors need to be controlled. We controlled for demographic explanatory (independent) variables as individual characteristics; these included *age*, *disability*, *marital status*, *education level*, *employment status*, and *income level*. A *gender* dummy variable was included as a time-constant variable that does not change over time. We used these variables to empirically analyze whether residential instability adversely affects the subjective health status and mental health of renters.

### 3.2. Analytic Approach

In this study, we analyzed the effect of residential instability on the subjective physical health status and overall depression of renters by performing a panel analysis using STATA 15.0 (STATA Corporation, College Station, TX, USA). First, we performed descriptive analysis and chi-squared or t-tests to compare renters with and without the experience of residential instability. Following this, a panel analysis using a fixed effects model was performed to investigate the effect of residential instability on health-related variables of household members. Using panel data, the total variance can be analyzed in terms of both within-variation and between-variation for each individual, which may result in autocorrelation and heteroskedasticity in the error term. Since the estimator of the ordinary least square (OLS) method does not meet the consistent estimates in the linear regression model, a generalized least square method must be used to calculate an efficient estimator [50]. An advantage of panel analysis is that it takes endogeneity issues into account. Specifically, it can help deal with the issue of endogeneity by removing time-constant, unobserved, and individual heterogeneity [51]. Equation (1) shows the basic formula for the panel analysis [52].

(1) $$Y_{i,t}=\alpha +X_{i,t}\beta +\epsilon_{i,t}$$

Here, *Y_i,t_* is perceived health status, which includes physical and mental health status; *i* represents the individual over time *t*, and *X_i,t_* represents the characteristics of residential security, welfare program, residential environments, and household members (individual). ϵ*_i,t_* represents *μ_i_* + λ*_t_* + ν*_i,t_*: *μ_i_* is the unobservable individual effect (differences between individual characteristics, but the variables remained constant over time); λ*_t_* is the unobservable individual effect (no difference between individual characteristics, and variable changed over time); ν*_i,t_* is a disturbance term that represents the difference between individual characteristics and changed variables over time. We conducted Hausman tests to estimate the degree to which unobserved individual differences are correlated with model covariates, and fixed effects specification is often more appropriate in many cases of within-variation of individuals. In this study, we can identify the effect of within-individual changes, such as having no experience of residential instability, on the within-variation of individuals. By using fixed effects, it is also possible to determine the effect of “intra-individual change”, such as the presence or absence of housing instability, which occurs from a single household member, against housing instability, which is an independent cause in this study.

## 4. Results

### 4.1. Descriptive Statistics for Renters with and without Experience of Residential Instability

Chi-square tests and t-tests were performed to compare the descriptive statistics of both groups and to identify differences in their subjective physical health, mental health, residential security, participation in welfare programs, residential environment, and individual characteristics. Table 3 compares the basic statistics of the two groups. Of the 28,887 samples (observations), 7.27% (*n* = 2101) were categorized into the residential instability group.

In terms of dependent variables, the residential stability group perceived themselves to be healthier (mean = 3.409, standard deviation (SD) = 1.057) and less depressed (mean = 15.913, SD = 5.381) compared to the residential instability group (mean = 3.121, SD = 1135; mean = 19.302, SD = 6.412, respectively). 

The residential instability group (75.54%) had a higher percentage of respondents living in houses that met the minimum housing standards than the residential stability group (45.41%). Additionally, there was a greater proportion (20.13%) of respondents living in (semi) basement and rooftop or non-residential housing in the residential instability group. Regarding the housing type, a greater proportion of respondents in the residential instability group (10.38%) lived in permanent rental housing compared to those in the residential stability group (9.03%), despite the benefits of housing assistance. The percentage of renters with residential instability and national health insurance (73.20%) was lower than that of respondents with stable housing (77.53%). The proportion of respondents with national basic life security was higher in the residential instability group (32.79%) than in the residential stability group (28.58%).

Compared to the residential stability group, the residential instability group tended to be dissatisfied with their residential environment (2.610) and social relationships (3.380). The majority of the residential instability group lived in the Seoul metropolitan area (55.16%).

A higher proportion of respondents in the residential stability group were employed (55.26%) compared to those in the residential instability group (51.19%). The education level of the residential instability group (19.51% or higher) was slightly lower than that of the residential stability group (28.03% or higher). Furthermore, the monthly personal income (KRW 612,860) was also lower for renters with residential instability. In terms of the number of household members, the residential instability group had more household members (2.805) than those in the residential stability group (2.640).

### 4.2. Fixed Effect Panel Model Results

Table 4 shows the results of the fixed effect model analysis of rental household members. The dependent variable in Model 1 was subjective physical health and that in Model 2 was overall depression. While an F-test was used for validation, taking into account individuals’ attributes with fixed error terms, a Breusch–Pagan test was used to examine the validity between pooled OLS and stochastic effects. The results showed that all panel models were more suitable than the pooled OLS model. In addition, the Hausman test was performed to select an appropriate model from the random effect model and the fixed effect model. The fixed effect model was found to be suitable for both Model 1 and Model 2. The effects of key variables in this study, including residential instability, on perceived health status must be interpreted based on the results of the fixed effect model.

According to the results of Model 1, residential instability, depression, national health insurance, and satisfaction with residential environment and social relationships had significant effects on the subjective physical health of renters after controlling for the effects of sociodemographic and other health-related (depression) factors. Specifically, higher levels of depression and residential instability had negative (−) effects on subjective health. However, living in houses that did not meet the minimum housing standards did not affect physical health. The results of Model 1 also showed that national health insurance had a positive (+) effect on health status. A higher satisfaction with residential environment and social relationships also had a positive (+) influence on health. These results are consistent with those of previous studies, which showed that the higher the degree of satisfaction with residential environment, the better the health status. Some of the sociodemographic variables also had an impact on subjective physical health. In particular, young adults, those without a disability, and employed individuals had a better health status, while the level of income did not significantly affect subjective physical health.

In Model 2, lower subjective physical health and residential instability increased the overall depression level after controlling for all individual, household, and health-related (subjective physical health) factors. Moreover, failure to meet the minimum housing standards is one of the factors that worsened depression in renters. Despite assistance benefits from the government, permanent rental housing and the national basic livelihood security also worsened depression. Dissatisfaction with residential environment and social relationships further increased overall depression. While income did not have a significant influence on overall depression, other characteristics, such as old age, unemployment, and being separated or divorced, also worsened depression.

## 5. Conclusions

In this study, we compared renters who experienced residential instability and those who did not based on a representative Korean sample surveyed from 2006 to 2019. In addition, we investigated the impact of renters’ housing problems on their perceived health status, including subjective health and mental health. This study addressed a limitation in previous studies by simultaneously considering the minimum housing standards and residential instability and their association with health outcomes among renters. Classifying renters at risk of residential instability into detailed types and examining the impact of residential instability is not possible due to limited data; however, all individuals who experienced residential instability were forced to move out because of difficulties making rent payments or overdue rent for two months or more. Due to frequent residential moves among households experiencing residential instability, recruiting samples and constructing datasets were difficult. Moreover, the sample size was not large enough to analyze. However, using longitudinal data from 14 waves of the KoWePS, we were able to investigate the causal relationship between residential instability and the perceived health status of renters. In addition, we also explored their sociodemographic characteristics and the role of residential instability in promoting the health status of renters.

The results of the analyses showed that most of the renters did not experience residential instability—only about 7% (*n* = 2101) did. The proportion of individuals experiencing residential instability was higher among renters living in substandard housing conditions, such as those that did not meet the minimum housing standards (75.54%) or (semi) basement, rooftop, or non-residential housing (20.13%). Renters who experienced residential instability perceived their health status and mental health to be relatively lower than those who did not experience residential instability. The results of the panel model analysis also showed that residential instability significantly affects both subjective health status and overall depression. As noted in the previous empirical findings, the results of this study show that common social disadvantages, such as age and employment, are related to residential instability and health-related variables [53]. One interesting finding is that the level of income did not affect both the subjective health status and overall depression. The level of income may have an indirect effect on health status only through residential instability. This indicates that residential instability may increase employment instability, which negatively affects one’s ability to live in a healthy residential environment.

Based on the findings of this study, it can be suggested that a high-quality living environment and satisfaction with social relationships may positively affect renters’ perceptions of their subjective physical health status and reduce depressive symptoms. Further, social exclusion of individuals in permanent rental housing may increase the feeling of isolation and aggravate their mental health risks. While permanent rental housing may contribute to reducing involuntary residential mobility for low-income renters, it may have a negative influence on their psychological health.

Interestingly, living in substandard housing did not appear to lead to significant deterioration of the physical health status of renters; however, it increased their overall depression. This is contrary to the results from previous studies, which reported that a poor residential environment could adversely affect one’s health. This may be suggested that anxiety about social disorder and being a victim of crime because of social isolation, poor quality, or lack of facilities in the living environment could significantly affect renters’ mental health and cause mental health disorders, such as stress and depression. 

Residential instability is highly related to housing affordability. Because tenants are relatively vulnerable to rising rents, they are more likely to experience residential instability than those living in owner-occupied homes. Rent payment or concerns about rent payment are often associated with mental fatigue. A recent study found that involuntary residential moves due to gentrification are highly associated with mental health deterioration, emergency room visits, and hospitalization [15]. Frequent residential mobility due to renters’ involuntary moves or residential instability may cause health-related problems during the process of seeking residential stability. Tenants who experience residential instability may have to quickly move out again, and they often face difficulties finding a reasonable living space due to limited resources and information, which, in turn, may result in choosing a poor residential environment. Furthermore, repeated risk of eviction may lead to living in poor living conditions. In addition, this train of thought may leave one with the impression that such renters are likely to experience difficulties in building and developing relationships and forming attachments with their neighbors.

Considering neighborhood effects of the sample families benefiting from welfare services in Korea, the survey should have included neighborhood-level factors that may contribute to health-related variables and residential instability. This limitation prevents us from considering local context variables. Nevertheless, the results of this study emphasize the need to pay attention to not only those who do not have a home to live in but also those who experience residential instability. For most Koreans, self-owned housing accounts for the majority of their wealth. Tenants who suddenly lose their homes or move out of their living space experience problems with bad credit and may have more difficulties achieving stable residential circumstances in the future [54].

Currently, Korean tenants, including residents of permanent rental housing, are not guaranteed protection from forced eviction because of overdue rent. Once they become homeless, the social costs of recovering and managing residential stability are likely higher than the costs of preventing residential insecurity. Given that concerns and displacement due to financial issues are the main difficulties of renters who experience residential instability, the most effective policy to reduce involuntary mobility might be increasing the supply of affordable housing. More public housing (permanent rental housing in the case of Korea) should be made available, with guidelines mandating regional developers to provide affordable housing, encouraging a fixed rate of rent increase and long-term leases from landlords. In addition to the quantitative supply of affordable housing, the overall quality of housing welfare services, including a focus on the mental health of low-income renters, needs to be improved by expanding the range of services and the number of professional housing welfare workers. To increase residential and social relationship satisfaction, local governments need to supply community facilities such as community centers to encourage social interaction among residents, which would also contribute to improving mental health by reducing social isolation. Furthermore, pilot programs, such as the Homelessness Prevention and Rapid Re-housing programs implemented in the United States, should also be initiated to examine whether such programs can improve the lives of low-income tenants who experience residential instability in order to elucidate the potential effectiveness of these anti-poverty housing programs. 

These suggestions can be a good solution for countries where the economic gap among residents is deepening. If governments pursue policies to maintain social stability based on these suggestions, it will reduce social problems caused by the economic gap.

In this study, we focused on the possibility of persistent stress caused by overdue rent and forced eviction, which, in turn, may result in the deterioration of perceived health status. However, due to the limitation of data availability, it was not possible to investigate which types of residential instability would have a major impact on the perceived health status of renters. Moreover, whether subjective health is an effective proxy for actual health status is debatable (see the review in Gunasekara, Carter, and Blakely [55]). Therefore, when relevant data are available, future studies should investigate the different types of residential instability and their effects on health-related variables (perceived and actual health status). This study conducted an empirical analysis of the effects of residential environments, which is recognized as a significant factor in low-income renters’ quality of life regarding their perceived health status. However, the various physical environments, including elements such as housing structure and neighborhood amenities, which were not included as part of the residential environments considered in this study, may also have a significant impact on health. Although the KoWePS did not include these types of environments and, therefore, they could not be included in the empirical analysis, if such environments can be used as control variables and key variables in the future, it will be possible to identify more diverse aspects affecting the health of low-income renters.

## Figures and Tables

**Table 1:** Depression index questionnaire.

Items	Rarely	Sometimes	Often	Mostly
(a) I did not want to eat; I had no appetite.	1	2	3	4
(b) It was relatively good.	1	2	3	4
(c) I was very depressed.	1	2	3	4
(d) Everything felt hard.	1	2	3	4
(e) I lost sleep.	1	2	3	4
(f) I felt the loneliness of being alone in the world.	1	2	3	4
(g) I was without major dissatisfaction.	1	2	3	4
(h) People were somewhat cold to me.	1	2	3	4
(i) I was sad.	1	2	3	4
(j) People did not like me.	1	2	3	4
(k) I did not want to do anything at all.	1	2	3	4
Depression Index	Sum of item scores (a–k)			

Source: The Korean Welfare Panel Study (KoWePS), waves 1–14.

**Table 2:** Variable description.

Category	Variable	Measurement
Dependent	Subjective health status	1 (poor)–5 (excellent)
	Overall depression	Sum of 11 item scores
Residential Security	Residential instability	0 = met 1 = unmet
	Minimum housing standards	0 = no 1 = yes
	Non-residential housing	0 = no 1 = yes
Welfare Program	Permanent rental housing	0 = no 1 = yes
	National health insurance	0 = no 1 = yes
	National basic living security	0 = no 1 = yes
Residential Environment	Satisfaction with residential environment	1 (very dissatisfied)–5 (very satisfied)
	Satisfaction with social relationships	1 (very dissatisfied)–5 (very satisfied)
	Metropolitan area	0 = no 1 = yes
Individual Characteristics	Gender	0 = female 1 = male
	Age	years
	Disability	0 = non-disabled 1 = disabled
	Marital status	0 = single 1 = married 2 = separated/divorced
	Education level	0 = elementary or less 1 = high school or less 2 = college or higher
	Employment status	0 = no 1 = yes
	Income level	Monthly household disposable income (10,000 won)/number of household members

Source: The Korean Welfare Panel Study (KoWePS), waves 1–14.

**Table 3:** Descriptive statistics for renters with and without residential instability.

Variables	Total Mean (Standard Deviation) *N* = 28,887	Residential Stability *N* = 26,786	Residential Instability *N* = 2101
Subjective health status (5-point Likert) ***	3.388 (1.066)	3.409 (1.057)	3.121 (1.135)
Depression index (number) ***	16.160 (5.533)	15.913 (5.381)	19.302 (6.412)
Minimum housing standards (%) ***	47.60	45.41	75.54
Non-residential housing (%) ***	9.84	9.03	20.13
Permanent rental housing (%) *	9.13	9.03	10.38
National health insurance (%) ***	77.21	77.53	73.20
National basic life security (%) ***	28.89	28.58	32.79
Residential satisfaction (5-point Likert) ***	3.133 (0.938)	3.174 (0.920)	2.610 (1.005)
Social relationship satisfaction (5-point Likert) ***	3.561 (0.775)	3.575 (0.762)	3.380 (0.896)
Metropolitan area (%) **	44.20	43.34	55.16
Gender (%) *	43.10	42.93	45.26
Age (years) *	48.881 (17.861)	48.951 (17.981)	47.980 (16.227)
Disability (%)	12.94	12.91	13.37
Marital status (%) ***			
Single (yes = 1)	20.40	20.60	19.80
Married (yes = 1)	47.43	47.60	45.17
Separated/Divorced (yes = 1)	30.18	29.80	35.03
Education level (%) ***			
Elementary or less (yes = 1)	25.45	25.21	28.46
High school or less (yes = 1)	47.15	46.76	52.02
College or higher (yes = 1)	27.41	28.03	19.51
Employment (%) ***	54.96	55.26	51.19
Income level (KRW 10,000) ***	99.969 (129.612)	103.004 (133.640)	61.286 (40.853)

Note 1: * *p* < 0.05; ** *p* < 0.01; *** *p* < 0.001. Source: The Korean Welfare Panel Study (KoWePS), waves 1–14.

**Table 4:** Results of the fixed effect panel model.

Variables	Model 1	Model 2
Dependent variable	Subjective physical health	Depression index
Subjective physical health	-	−0.073 (0.004) ***
Depression index	−0.256 (0.012) ***	-
Residential instability	−0.080 (0.020) ***	0.101 (0.010) ***
Minimum housing standard	−0.010 (0.012)	0.024 (0.006) ***
Non-residential housing	0.015 (0.024)	0.001 (0.013)
Permanent rental housing	−0.038 (0.023)	0.027 (0.012) *
National health insurance	0.130 (0.026) ***	−0.002 (0.014)
National basic life security	−0.040 (0.024)	0.047 (0.013) ***
Residential satisfaction	0.048 (0.006) ***	−0.036 (0.003) ***
Social relationship satisfaction	0.035 (0.007) ***	−0.081 (0.004) ***
Metropolitan area	−0.068 (0.048)	−0.003 (0.026)
Gender	Omitted	Omitted
Age	−0.008 (0.002) ***	−0.015 (0.001) ***
Disability	−0.110 (0.046) *	0.032 (0.025)
Marital status (reference = single)		
Married	−0.001 (0.051)	0.023 (0.027)
Separated/divorced	−0.062 (0.054)	0.071 (0.029) *
Education level (reference = elementary or less)		
High school or less	−0.090 (0.081)	0.044 (0.043)
College or higher	−0.116 (0.102)	0.053 (0.055)
Employment	0.090 (0.015) ***	−0.030 (0.008) ***
Income level	0.001 (0.001)	−0.001 (0.001)

Note 1: * *p* < 0.05; ** *p* < 0.01; *** *p* < 0.001. Note 2: Both models 1 and 2 Prob > x^2^ = 0.000. Note 3: Fixed effect model is more suitable based on the results of Hausman tests (*p* < 0.01). Source: The Korean Welfare Panel Study (KoWePS), waves 1–14.
